# Phytosterols in the Treatment of Hypercholesterolemia and Prevention of Cardiovascular Diseases

**DOI:** 10.5935/abc.20170158

**Published:** 2017-11

**Authors:** Carlos Eduardo Cabral, Márcia Regina Simas Torres Klein

**Affiliations:** 1 Divisão de Nutrição - Hospital Universitário Pedro Ernesto - Universidade do Estado do Rio de Janeiro (UERJ), Rio de Janeiro, RJ - Brazil; 2 Departamento de Nutrição Aplicada - Instituto de Nutrição - Universidade do Estado do Rio de Janeiro, Rio de Janeiro, RJ - Brazil

**Keywords:** Cardiovascular Diseases, Phytosterols, Atherosclerosis, Cholesterol, LDL

## Abstract

Phytosterols are bioactive compounds found in foods of plant origin, which can be divided into plant sterols and plant stanols. Clinical studies consistently indicate that the intake of phytosterols (2 g/day) is associated with a significant reduction (8-10%) in levels of low-density lipoprotein cholesterol (LDL-cholesterol). Thus, several guidelines recommend the intake of 2 g/day of plant sterols and/or stanols in order to reduce LDL-cholesterol levels. As the typical western diet contains only about 300 mg/day of phytosterols, foods enriched with phytosterols are usually used to achieve the recommended intake. Although phytosterols decrease LDL-cholesterol levels, there is no evidence that they reduce the risk of cardiovascular diseases; on the contrary, some studies suggest an increased risk of atherosclerosis with increasing serum levels of phytosterols. This review aims to address the evidence available in the literature on the relationship between phytosterols and risk of cardiovascular disease.

## Introduction

Cardiovascular diseases (CVD) remain the main cause of mortality worldwide, accounting for about 30% of all deaths.^1^ During the last two decades, deaths due to CVD decreased in developed countries but increased sharply in low- and middle-income countries.^1,2^

Atherosclerosis is the main pathological process that leads to the development of CVD, including acute myocardial infarction (AMI), heart failure, and stroke.^3^ Early identification of risk factors for CVD is fundamental for the prevention of the onset and/or progression of atherosclerosis. The main risk factors for atherosclerosis include smoking, hypertension, diabetes mellitus, advanced age, family history of CVD, and dyslipidemia.^4,5^

The fundamental role of dyslipidemia - especially hypercholesterolemia - in the development of CVD has already been confirmed.^4^ Through a wide range of plasma cholesterol concentrations, there is a strong positive linear correlation between risk of CVD and levels of total cholesterol and low-density lipoprotein cholesterol (LDL-cholesterol) levels. Additionally, it has been demonstrated that a reduction in LDL-cholesterol levels reduces the risk of CVD.^4,6,7^

There is evidence that elevated serum LDL-cholesterol levels may cause atherosclerotic CVD independently of other risk factors.^8^ Dyslipidemia can be considered a primary risk factor for atherosclerotic CVD and may be a prerequisite for atherosclerosis, occurring before the participation of other risk factors.^5^ The increase in serum LDL-cholesterol concentrations appears to be necessary for atherogenesis. LDL comprises more than 75% of the atherogenic lipoproteins, with the remaining including cholesterol-rich remnants of lipoproteins rich in triglycerides (chylomicrons and very-low-density lipoproteins; VLDL). When LDL infiltrates into the arterial wall, it initiates and promotes atherosclerosis.^8^

According to the World Health Organization (WHO), 39% of adults (> 25 years) worldwide have increased total cholesterol concentrations (> 190 mg/dL). The prevalence is greater in Europe (54%), followed by the Americas (48%).^9^

The treatment of hypercholesterolemia must include nonpharmacological measures, which are recommended for all patients, as well as the use of pharmacological therapy that may be indicated in specific situations.^10^ The drugs currently available for the treatment of hypercholesterolemia include statins (hydroxy-methylglutaryl-coenzyme A [HMG-CoA] reductase inhibitors), ezetimibe (a selective inhibitor of cholesterol absorption), and resins or bile acid sequesters. Statins should be used as the first choice due to their powerful effect on LDL-cholesterol reduction (25-55%) and because they are the most study-validated drugs for reduction of cardiovascular events. Ezetimibe has a moderate effect on LDL-cholesterol reduction (15-25%). Resins may be associated with statins when the target LDL-cholesterol is not achieved despite the use of statins, leading to a reduction of 30% in LDL-cholesterol levels.^4,8,10^

Nonpharmacological treatment of dyslipidemia must include changes in dietary habits and physical activity, in addition to weight loss, when indicated.^10^ In the nutritional approach, intake of saturated fatty acids and trans fatty acids must be limited as well as the intake of cholesterol, in addition to increasing the intake of soluble fiber. The consumption of phytosterols is also indicated for the treatment of hypercholesterolemia, according to several guidelines and consensuses of different societies worldwide.^4,5,8,10-12^ There is consistent evidence that the intake of phytosterols (2 g/day) is associated with a significant reduction in LDL-cholesterol (8 - 10%).^11^ However, there are no data indicating that the consumption of phytosterols may reduce the risk of CVD. On the contrary, some studies suggest that the concomitant elevation in plasma concentration of phytosterols can increase the risk of development of atherosclerosis.^13,14^

This review aims at addressing the evidence available in the literature on the relationship between phytosterols and risk of CVD.

### Phytosterols

#### Definition, classification, and food sources

The term "phytosterols" is used to describe plant sterols and their saturated derivatives, plant stanols.^15,16^ Phytosterols are bioactive compounds found naturally in foods of plant origin and present a chemical structure similar to that of cholesterol,^17^ which is found only in foods of animal origin. More than 250 phytosterols have already been identified.^15^ The plant sterols more commonly found in the diet are beta-sitosterol, campesterol, and stigmasterol. In regards to plant stanols, beta-sitostanol, and campestanol are the two most common types.^18^

Food sources of phytosterols include vegetable oils, mainly corn (909 mg/100 mL), sunflower (411 mg/100 mL), soybean (320 mg/100 mL), and olive (300 mg/100 mL); oleaginous fruits such as almonds (183 mg/100 g); cereals like wheat germ (344 mg/100 g), and wheat bran (200 mg/100 g); in addition to fruits and vegetables, such as passion fruit (44 mg/100 g), orange (24 mg/100 g), and cauliflower (40 mg/100 g).^18^ A typical western diet contains approximately 300 mg of sterols and 30 mg of plant stanols,^17^ while vegetarian diets can achieve a higher content (300 - 500 mg/day).^18^ This amount of phytosterols present in a regular diet is considered small to achieve the recommended daily intake of phytosterols able to present therapeutic effects on LDL-cholesterol reduction (~2 g/day), and it is generally required the consumption of foods enriched with phytosterols or, alternatively, the use of supplements of phytosterols.^16,18^ In Brazil, some processed foods enriched with phytosterols are available, including margarine, yogurt, and milk.

#### Mechanism of action on cholesterol

The main mechanism by which phytosterols lower LDL-cholesterol levels is through a reduction (30 - 50%) in the intestinal absorption of cholesterol.^15,16^ This reduction may be mediated by some mechanisms, in particular, the competition with cholesterol by the solubilization in mixed micelles in the intestinal lumen, reducing the amount of cholesterol available for absorption.^15,19^ Other proposed mechanisms include: (1) modification in the expression of genes that encode the proteins that carry sterols, such as the Niemann-Pick C1-like 1 (NPC1-L1) protein, reducing the transport of cholesterol to the enterocyte, or ATP-binding cassette transporters (ABCG5 and ABCG8), promoting the efflux of cholesterol from the enterocytes to the intestinal lumen; (2) reduced rate of cholesterol esterification in the enterocyte; and (3) increased removal of cholesterol from the body through the transintestinal cholesterol excretion (TICE).^17^ In response to a decrease in the absorption of dietary cholesterol, hepatic synthesis of cholesterol seems to increase, but the increase in hepatic release of cholesterol is not sufficient to compensate for the lower absorption of dietary cholesterol.^16^

Intestinal absorption of plant sterols (< 2%) and stanols (< 0.2%) is much lower than that of cholesterol (~50%).^17^ As a result of the low absorption and efficient biliary excretion after hepatic uptake, circulating levels of phytosterols are very low, ranging from 0.3 to 1.0 mg/dL for plant sterols and 0.002 to 0.012 mg/dL for plant stanols. The distribution of phytosterols through the main classes of lipoproteins is similar to that of cholesterol; therefore, they circulate mainly in LDL particles (65 - 70%).^11^

### Cholesterol-lowering effect of phytosterols

Since the late 1950s, numerous studies have consistently indicated that foods enriched with phytosterols reduce the concentrations of LDL-cholesterol.^20-24^ Table 1 shows some randomized, placebo-controlled clinical trials published since 2010 that evaluated the effects of foods enriched with phytosterols on the cholesterolemia. The Table demonstrates that the dose is around 2 - 4 g/day and the reduction in LDL-cholesterol is ~10%. For example, the study by Párraga-Martínez et al.^25^ evaluated the intake of 2 g/day of plant stanols during 12 months and observed an 11% reduction in LDL-cholesterol levels. The study of Vásquez-Trespalacios & Romero-Palacio (2014)^26^ evaluated the intake of 4 g/day of plant stanols and observed after 4 weeks a 10.3% decrease in LDL-cholesterol levels. Among the studies listed in Table 1, the study that used the highest dose of phytosterols (8.8 g/day of plant stanols) was the one conducted by Gylling et al. (2010),^27^ which showed a 17.1% reduction in LDL-cholesterol levels.

**Table 1:** Randomized clinical trials evaluating the effects of supplementation of phytosterols on cholesterolemia

Authors (year)	n	Type of phytosterol (food/supplement)	Dose of phytosterol (g/day)	Duration	↓ LDL-cholesterol (%)	P value (*versus* control group)
**Studies with foods enriched with phytosterols**						
Gylling et al. (2010)^27^	49 individuals with mild-to-moderate hypercholesterolemia	Stanols (spread and drink)	8.8	10 weeks	17.1	0.01
Gylling et al. (2013)^39^	92 asymptomatic individuals (not using lipid-lowering drugs)	Stanols (spread)	3	6 months	10.2	0.001
Buyuktuncer et al. (2013)^40^	70 individuals with mild-to-moderate hypercholesterolemia	Stanols (yogurt)	1.9	4 weeks	6.3	0.005
Vásquez-Trespalacios & Romero-Palacio (2014)^26^	40 individuals with moderate hypercholesterolemia	Stanols (yogurt)	4	4 weeks	10.3	< 0.01
Ras et al. (2015)^41^	240 individuals with hypercholesterolemia	Sterols (spread)	3	12 weeks	6.7	< 0.05
Párraga-Martinez et al. (2015)^25^	182 adults with hypercholesterolemia	Stanols (yogurt)	2	12 months	11	0.01
**Studies with capsules or tablets **						
Maki et al. (2012)^32^	32 subjects with primary hypercholesterolemia	Sterols/Stanols (pill)	1.8	6 weeks	4.9	< 0.05
Maki et al. (2013)^29^	28 subjects with primary hypercholesterolemia	Sterols/Stanols (softgel capsule)	1.8	6 weeks	9.2	< 0.001
Ottestad et al. (2013)^30^	41 individuals with total cholesterol 180 - 300 mg/dL	Sterols/Stanols (softgel capsule)	2	4 weeks	2.7	0.32
McKenney et al. (2014)^31^	30 adults with familial hypercholesterolemia	Sterols/Stanols (softgel capsule)	1.8	6 weeks	4.3	< 0.01

LDL-cholesterol: low-density lipoprotein cholesterol.

Recent meta-analyses confirmed the cholesterol-lowering effect of phytosterols, in addition to comparing the effects of sterols with those of stanols, and evaluating the dose-response relationship.^21-24^ The dose-response relationship presented a slight variation among the meta-analyses, and there is still no consensus. A meta-analysis conducted by Ras et al.^24^ included 124 studies with a mean phytosterol dose of 2.1 g/day (range 0.2 to 9.0 g/day). The intake of 0.6 - 3.3 g/day was associated with a gradual decrease in the concentration of LDL-cholesterol of 6 - 12%. Plant sterols and stanols had comparable effects. The studies with doses exceeding 4 g/day were not grouped together, because they were few and had large dose variations. The authors concluded that the LDL-cholesterol reducing effect of both plant sterols and stanols increased until an intake of approximately 3 g/day, with a mean effect of 12%.^24^ A meta-analysis conducted by Talati et al.^22^ compared the effects of plant sterols and stanols on LDL-cholesterol and also observed no significant differences.

The data indicating the cholesterol-lowering effect of foods enriched with phytosterols derive from intervention studies with good methodological quality, conducted with a relatively large number of participants, and the results are generally consistent.^28^ According to several international scientific societies, the regular use of 2 g/day of phytosterols under supervision can be recommended for a 10% LDL-cholesterol reduction.^4,5,8,10-12^

Leaving the scenario of fortified foods, some recent studies have evaluated the effects of phytosterols in tablets or capsules,^29-32^ with the objective of evaluating whether this form of supplementation (considered more practical by many authors) would also be effective in lowering cholesterol. The study by Maki et al.^29^ used softgel capsules, providing 1.8 g/day of esterified sterols/stanols in conjunction with lifestyle changes recommended by the National Cholesterol Education Program (NCEP) for 6 weeks and observed a 9.2% reduction in LDL-cholesterol levels. A significant reduction in LDL-cholesterol levels was also observed in studies conducted by Maki et al.^32^ and McKenney et al..^31^ However, the study conducted by Ottestad et al.^30^ did not observe a significant reduction in LDL-cholesterol levels. A recent meta-analysis^33^ including eight studies published from 1992 to 2013 with a duration of 4 - 6 weeks and doses of phytosterols between 1 to 3 g/day in tablets or capsules observed a significant reduction in LDL-cholesterol (on average 12 mg/dL), which was similar to that observed with food enriched with phytosterols. Therefore, despite the lack of consensus, most studies indicate that the use of phytosterols in tablets or capsules can be effective in reducing LDL-cholesterol levels.

There is evidence that the consumption of phytosterols in association with lipid-lowering therapy is able to promote a further reduction in serum cholesterol levels. These benefits have been observed in association with statins^34-36^ and also with ezetimibe.^37,38^ The meta-analysis developed by Han et al.^36^ included 15 randomized clinical trials that evaluated the effect of diets enriched with phytosterols in patients using statins. The phytosterols in combination with statins, compared with statins alone, produced a significant reduction of 12 mg/dL in LDL-cholesterol levels.

### Phytosterols and cardiovascular disease

The direct relationship between intake of foods enriched with phytosterols and CVD risk has not been investigated in randomized clinical trials. It has been estimated that it would be necessary to follow up at least 33,000 individuals for about 10 years for a proper assessment of the effects of phytosterols on hard endpoints, hindering the viability of such studies.^42^

By inference based on the cardioprotective efficacy of other cholesterol-lowering interventions, some authors consider that phytosterols may reduce cardiovascular risk; however, this statement should not be performed until studies proving this fact are available.^43^

#### - Serum levels of phytosterols

The speculation in regards to a potential deleterious effect of phytosterols has been largely motivated by the fact that phytosterolemia (also known as sitosterolemia), a rare autosomal recessive disease, is characterized by a 50-fold increased circulating concentration of plant sterols and may be associated with early atherosclerosis. However, the consumption of foods enriched with phytosterols is associated with a much lower increase (around twice) in circulating plant sterols.^44^

Several studies have evaluated the association between plasma phytosterol concentration and CVD; however, the results are conflicting. Some studies have found a positive association between serum levels of phytosterols (or the relationship phytosterols/cholesterol) and risk of CVD;^13,14^ while others did not observe any association or even found an inverse association.^45-47^ Genser et al.^48^ published a systematic review and meta-analysis based on 17 studies involving 11,182 individuals and found no evidence of an association between serum concentration of phytosterols and development of CVD. The authors of this meta-analysis attributed the great divergence in the results of the studies to the different designs of studies and adjustments for potential confounding variables. They suggest that biases can occur if the investigators fail to make appropriate adjustments, mainly for serum levels of lipoproteins, in particular for LDL-cholesterol.^48^ Another possibility is the fact that circulating phytosterols alone do not increase the risk of CVD but are rather only markers of cholesterol absorption.^49^

#### - Intermediate markers of cardiovascular risk

Due to the absence of studies evaluating cardiovascular outcomes, the investigation of intermediate risk markers for CVD represents an acceptable and viable form to evaluate the relationship between phytosterols and cardiovascular risk. Currently, there are available studies assessing endothelial dysfunction, arterial stiffness, diameter of the retinal vessels, and inflammation of low degree.^39,41,50-54^

In a study conducted by Gylling et al.,^39^ the consumption of plant stanols (3 g/day) for 6 months showed beneficial effects on arterial stiffness, especially in men. In addition, endothelial function, assessed by peripheral arterial tonometry, improved with a reduction in LDL-cholesterol and non-high-density lipoprotein (non-HDL) cholesterol. A study conducted by Heggen et al.^52^ tested the effects of two margarines enriched with plant sterols (2 g/day) from two different vegetable oils during 4 weeks. The authors observed a significant reduction in E-selectin and plasminogen activator inhibitor 1 (PAI-1) with the ingestion of only one of the margarines. In this study, there was no significant reduction in vascular cellular adhesion molecule-1 (VCAM-1) and tumor necrosis factor alpha (TNF-α) with any of the margarines, and also no association was observed between LDL-cholesterol reduction and changes in E-selectin and total PAI-1.^52^

On the other hand, supplementation with 3 g/day of plant sterols for 12 weeks in 240 subjects with hypercholesterolemia did not result in beneficial effects on arterial stiffness and endothelial function.^41,54^ In this study, the endothelial function was assessed by flow-mediated dilation^41^ and circulating biomarkers: intercellular adhesion molecule-1 (ICAM-1), VCAM-1, and E-selectin.^54^

In two crossover clinical trials including children with familial hypercholesterolemia, the consumption of phytosterols during 4 weeks failed to improve endothelial function assessed by flow-mediated dilation, although it induced a significant reduction in LDL-cholesterol levels.^50,51^

A randomized clinical trial conducted by Kelly et al.^53^ evaluated the effects of consumption over the long term (85 weeks) of plant sterols and stanols on the diameter of retinal vessels (microcirculation). The study included three groups of patients who consumed margarine enriched with plant sterols (2.5 g/day), margarine enriched with plant stanols (2.5 g/day), and margarine without phytosterols. There were no significant changes in venular diameter in the three groups, but the changes in serum concentrations of campesterol (a type of plant sterol) were positively associated with the changes in venular diameter, regardless of LDL-cholesterol level (r = 0.39, p = 0.03).

In regards to inflammation, there was no significant reduction in any of the markers evaluated in the study conducted by Ras et al.^54^: C-reactive protein (CRP), serum amyloid A, interleukin (IL)-6, IL-8, TNF-α, and ICAM-1. In a systematic review and meta-analysis recently published, Rocha et al.^55^ evaluated the effect of consumption of phytosterols on inflammatory markers, particular on CRP. The study included 20 randomized clinical trials (n = 1,308) involving foods enriched with phytosterols as active treatment. The reduction in CRP concentration with the consumption of phytosterols was 0.10 mg/dL, which did not reach statistical significance.^55^

The results of these studies evaluating the effects of phytosterols on intermediaries markers of cardiovascular risk observed no consistent beneficial effects. Thus, there is no current evidence that the use of phytosterols may reduce the risk of CVD by acting on these markers.

#### - Intake of oxidized phytosterols

Recent publications have alerted to another potential deleterious effect of phytosterols: the intake of oxidized phytosterols. Plant sterols (but not stanols, because they are saturated) may oxidate, forming oxidized phytosterols and, similarly to what is observed with cholesterol oxidation, these substances are believed to be atherogenic.^56^ However, in studies with humans, there is still no consensus on whether the intake of foods enriched with sterols is able to increase the serum concentration of oxidized phytosterols. For example, in the clinical randomized, crossover trial conducted by Baumgartner et al. (2013),^57^ 43 healthy individuals consumed during 4 weeks margarine enriched with sterols (3 g/day), margarine enriched with stanols (3 g/day), and a control margarine. The consumption of margarine enriched with sterols did not increase the serum concentration of oxidized phytosterols.^57^ Another study by the same group^58^ investigated the effects of intake of sterols on the concentration of oxidized phytosterols during the postprandial period. In this study, the individuals consumed a drink containing none or 3 g of plant sterols or stanols. Blood samples were collected for up to 8 h, and 4 hours later, the individuals received a second drink (without sterols or stanols). The concentration of oxidized phytosterols increased significantly after consumption of the meal with sterols in comparison with the meal with stanols and the control meal. This increase was only observed after the consumption of the second drink and the authors concluded that it is still unclear whether the increase in oxidized phytosterols in the postprandial period is due to absorption or endogenous formation. Therefore, until the present moment, there is no consensus on the role of oxidized phytosterols in the development of CVD.

### Phytosterols and liposoluble vitamins

Considering that phytosterols reduce the intestinal absorption of cholesterol, it is reasonable to imagine that these substances may also reduce the absorption of liposoluble vitamins and antioxidants. The serum levels of vitamins A, D, and K1 are generally not affected by the consumption of phytosterols.^59^ However, some studies suggest that phytosterols may promote a modest reduction in plasma concentration of carotenoids (mainly β-carotene, α-carotene, and lycopene)^27,60^ and tocopherols,^61^ but other studies have not observed this fact.^62,63^ A recently published meta-analysis evaluated the effects of phytosterol consumption in plasma concentrations of liposoluble vitamins and carotenoids. It included 41 randomized clinical trials (n = 3,306) with a mean phytosterol intake of 2.5 g/day. In the analyses adjusted for total cholesterol, there was a significant reduction in the concentration of hydrocarbon carotenoids (β-carotene, α-carotene, and lycopene) and some oxygenated carotenoids (zeaxanthin and cryptoxanthin). In contrast, there was no significant reduction in the concentration of tocopherol, vitamin D, or retinol. A very important finding of this meta-analysis was that the concentration of these substances remained within the normal range, giving no indication that the observed reductions could have negative health implications.^64^ Noakes et al.^65^have demonstrated that it is possible to avoid reductions in plasma carotenoid concentrations during the consumption of phytosterols through an increase in daily consumption of carotenoid-rich fruits and vegetables.

### Is the consumption of phytosterols safe?

There are still no available studies with long-term follow-up ensuring the safety of regular consumption of products enriched with phytosterols, as highlighted in recent publications of the European Society of Cardiology / European Atherosclerosis Society^12^ and the American Heart Association / American College of Cardiology.^66^ However, based on the absence of adverse effects in short-term studies and experimental studies, several authors consider that the consumption of phytosterols is safe and may be indicated for lowering cholesterol, including in association with drug therapy.^11,59,63,67,68^ Furthermore, different scientific societies recommended the use of phytosterols in the treatment of hypercholesterolemia.^4,5,8,10-12,28^ It is important to note that phytosterol supplementation is contraindicated in the rare patients presenting phytosterolemia (or sitosterolemia).^16,67^

The addition of phytosterols to industrialized food as an ingredient to reduce cholesterol has already been approved by several regulatory agencies around the world, including Health Canada, U.S. Food and Drug Administration (FDA), European Food and Safety Authority (EFSA), Food Standards Australia New Zealand (FSANZ),^16^ and National Health Surveillance Agency (ANVISA) in Brazil.^69^

According to ANVISA, foods enriched with phytosterols should display the following label information: "Phytosterols help reduce the absorption of cholesterol. Their consumption must be associated with a balanced diet and a healthy lifestyle." ANVISA determines that to display this information, the portion of the product ready for consumption should provide at least 0.8 g of free phytosterols. In addition, the label of these products should include phrases such as: "The product is not suitable for children younger than 5 years, and pregnant or nursing women."^69^

## Final considerations

The European Atherosclerosis Society recently published a consensus on phytosterols that concluded that based on the reducing effect of LDL-cholesterol and absence of adverse signs, the consumption of foods enriched with phytosterols may be considered: (1) in individuals with hypercholesterolemia presenting intermediate or low cardiovascular risk without indication of pharmacotherapy, (2) as an adjunct to pharmacological therapy in patients with high and very high cardiovascular risk who fail to achieve the goals of LDL-cholesterol with statins or are intolerant to statins, and (3) in adults and children (> 6 years) with familial hypercholesterolemia, along with lifestyle changes and drug therapy.^11^

Most guidelines and consensus on the treatment of dyslipidemia and/or prevention of CVD recommend the intake of phytosterols in the amount of approximately 2 g/day with the goal of reducing LDL-cholesterol by approximately 10%, in association with lifestyle changes.^4,5,8,10-12,28^

Currently, the knowledge about the relationship between consumption of phytosterols and risk of CVD is incomplete. The available evidence does not confirm that phytosterols may confer cardiovascular protection and also does not show deleterious effects. Further studies are needed, especially with long-term supplementation of phytosterols.
